# The experience of reading philosophy

**DOI:** 10.3389/fpsyg.2022.1019681

**Published:** 2022-10-20

**Authors:** Daniel Whistler

**Affiliations:** Department of Politics, International Relations and Philosophy, University of London, Egham, United Kingdom

**Keywords:** epistemic breakthrough, lay readers, philosophy as a way of life, passion, Martha Nussbaum, Immanuel Kant

## Abstract

Reading is not a peripheral philosophical pastime; it constitutes most of what we do when we do philosophy. And the experience of reading philosophy is much more than just a series of interpretative acts: the philosopher-reader is subject to, among other things, sensations, passions, emendations, and transformations. In this essay, I argue that a full account of philosophical reading should outline some of the sociological structures that determine how different communities of philosophers (within and outside the academy) construct such experiences, as well as describe in detail the ways in which philosophers encounter (or fail to encounter) truths while reading. It should, that is, describe ways in which philosophy acts upon readers and the various effects that result.

## Introduction

The phrase “experience of reading” used throughout this essay is informed by three distinct, if cognate contexts. First, it refers back to Hans Robert Jauss’ (1982: 153) excavation of the affects of reading, those “primary levels” of readerly enjoyment which include “astonishment, admiration, being shaken or touched, sympathetic tears and laughter, or estrangement.” I am particularly interested in Jauss’ insistence that pleasure and interpretation cannot be pulled apart in the reading process, i.e., in a process composed of “a completely sensuous and a highly intellectual affect” (1982: 23).^1^ Secondly, this notion of “the experience of reading” draws on Philip Davis’ *The Experience of Reading* (as well as much of his later work) which is, among other things, an attempt to perform in front of his reader the kinds of thinking that takes place as we read—what I will go on to call, “thinking-in-reading.” Davis (1991: 4) is interested in the eventhood of reading, in the fact that “something real goes on in the act of reading.” That is, he is interested in describing as precisely as possible the idea that “thought is something that occurs to a reader as if it were an event” (1991: xvi). In so doing, Davis takes up a notion of experience with roots in William James’ *The Varieties of Religious Experience* which itself describes the nuances of mood, intricacies of epiphany and relatively indeterminate ebbs and flows of consciousness involved in the constitution of (religious) meaning (James, 2008). Thirdly, the phrase “experience of reading” is taken from a passage in Martha Nussbaum’s essay, “Perceptive Equilibrium,” in which she compares “the experience of reading” Derrida’s book on Nietzsche, *Spurs* (Derrida, 1979), with that of reading Nietzsche himself. This experience of reading Derrida is, she writes (Nussbaum, 1992: 171), shot through with “an empty longing,” “a hunger” for some of the “difficulty,” “risk,” and “urgency” that can be found within Nietzsche’s own texts. “After reading Derrida,” Nussbaum concludes, “I feel a certain hunger for blood” (1992: 171).^2^

Overall, what these three different contexts are meant to start “getting at,” in a cumulative fashion, is a richer, even “thicker” account of reading philosophy than those accounts professional philosophers usually tell themselves and each other. What interests me in this essay are the features of this reading experience and their implications.

## An “off-duty” reading of the *Critique of Pure Reason*

I imagine (a far-flung idea!) that one weekend, at a moment of leisure free from all other obligations including any thoughts of research or teaching, I pick up Kant’s *Critique of Pure Reason* to read a few pages. This is not my first time reading it^3^, but it is a book that has no immediate relation to any of my current research projects or classes, and so I will not be mining it for selective arguments, concepts or contexts, but just turning its pages because I hope I will enjoy doing so. I could of course have chosen some fiction or popular non-fiction, but I actually like (or remember liking) philosophy—even Kant! As far as is possible, my professional “reading self” is muted—I am “off duty”^4^—and, instead, I desire to read a few pages of the *Critique of Pure Reason* “daring to behave like a deliberate amateur” (Davis, 1991: 22).

What might I (re)discover in the *Critique of Pure Reason*?^5^ I might—to take some obvious examples—simply mouth “wow!” at my initial thrill before the grandeur and scope of Kant’s proposed project in the B-edition Preface (e.g., Kant, 1998: Bxii–xvii); I might turn to the opening of the Transcendental Aesthetic (Kant, 1998: A19-21/B33-6) to experience a familiar sense of alienation at his insistence on redefining the basic terms of the philosophical tradition in jarring, often counterintuitive ways; or I might choose a much later, less forbidding entry-point, such as the first page of the section on “the ground of the distinction of all objects in general into phenomena and noumena” with its extended allegory of “the land of truth” and stormy sea of error (1998: A235-6/B294-5) and find myself smiling at the drama of it all, as well as remembering how much I loved such drama as an undergraduate.

Others’ “off-duty” reactions to the *Critique of Pure Reason* are not difficult to unearth either. At one end of the spectrum are those who encounter Kant’s text, like Robert Musil’s (2016: 505) General Stumm, having experienced its “*rigor mortis*,” its “geometric plague,” and conclude that they “do not want to go on reading,” or those who, like Joseph Joubert (1938: 297; translated in Nancy, 2008: 138), are put off by the first *Critique*’s “painful language”—as “painful for [Kant] to construct” as “it is painful to understand.” At the other end of the spectrum, though, are those whose personal experience of reading the *Critique of Pure Reason* is passionate, enthusiastic, even inspired by a sense of adventure—such as de Quincey’s (2003, 62) “opium-eater” who “read Kant again, and again understood him, or fancied that I did… [and] my feelings of pleasure expanded themselves to all around me,” or such as Ernst Horneffer who confesses,

I am unable to read the *Critique of Pure Reason* without feeling the most violent agitation. Every word in it, it seems to me, is incandescent, shot through with the frisson of the most profound, the truest, the most elementary feeling. No other poem seeking to communicate or the immediacy of feeling, except perhaps *Faust*, is able to produce an affective impression equal to the one I receive from this work, apparently glacial, of pure thought. As strange as it may seem, the *Critique of Pure Reason* is for me one of the most passionate, indeed the most passionate, of world literature. (1920: 67; translated in Nancy, 2008: 61–62)

At stake in all these experiences of the *Critique of Pure Reason* is something Horneffer makes very explicit: the readerly passions ineluctably felt when doing philosophy. That is, doing philosophy typically requires doing some reading (even if the amount and type of reading varies) and this reading process can be exciting, frustrating, surprising, boring, exhilarating, galling, gripping, dispiriting, even fun. It can also be transformative and informed by a sense of discovery: it can seduce us, resonate with us, alienate us, reprimand us and make us care about many new things (including the text in front of us). Nevertheless, professional philosophers tend to be fairly incurious about both analyzing these effects of reading on ourselves and on others and also incorporating them into academic reconstructions of acts of interpretation. This seems a shame: not only (as I’ve begun to suggest) are affective and transformative experiences common when reading philosophy, but they are also not obviously irrelevant to the project of truth-seeking, even in its most scholastic forms. Excitement, seduction and rapture often emerge entwined with those “aha-moments” (i.e., epistemic breakthroughs)^6^ which philosophers covet; and, correspondingly, frustration, alienation and the urge to quit signal a failure to “get it” that is, I’m going to contend, equally philosophically significant. All of them frequently take the form of what Daston and Park (1998) call `cognitive passions’ and what Morton (2010) calls `epistemic emotions’. In sum, the above experiences of reading philosophy matter when doing philosophy, even if most modern philosophers have tended to presume they do not.

## Pathologies of the philosopher-reader

There are, I want to postulate, at least three reasons why modern philosophers have tended to be so incurious about these sorts of experiences reading philosophy.^7^

### The “becoming-literature” of philosophy

Horneffer’s reaction to the *Critique of Pure Reason* is exemplary of the way reading philosophy can be a form of self-discovery. He speaks of a violence experienced on reading the first *Critique*, an emotional upheaval that stands in tension with Kant’s own will-to-abstraction. And this very personal experience in turn generates a personal set of critical categories by which Horneffer (1920: 67) tries to articulate this passionate revolution—terms such as “incandescence,” “frisson,” “affective impression.” And yet, within this passage such personal vocabulary comes at a price: the “reduction” of Kant’s book to a piece of “world-literature” which stands alongside Goethe’s *Faust* and other “poems.” Horneffer can only communicate his passionate experience of Kant’s text by treating it as literature; it is only outside philosophy that such passion finds a voice.

This is a common way by which philosophers immunize themselves against these kinds of experiences reading philosophy—by “othering” them into the domain of literature. Nussbaum’s account of her experience reading Derrida mentioned above (Nussbaum, 1992: 170-171) operates in a similar fashion. It forms part of an argument intended to demonstrate that a “sense of practical importance… is absent from the writings of many of our leading literary theorists,” To this end, Nussbaum writes, “One can have no clearer single measure of this absence than to have the experience of reading Jacques Derrida’s *Éperons* after reading Nietzsche… After reading Derrida, and not Derrida alone, I feel a certain hunger for blood; for, that is, writing about literature that talks of human lives and choices as if they matter to us all” (Nussbaum, 1992: 171). Again, Nussbaum’s experience is one of a personal, passionate reaction to Derrida’s text framed in the language of longing, hunger, risk and urgency. But, once again, this occurs at the expense of consigning Derrida to literary theory and Nietzsche to “literature.”^8^ To experience philosophy in this way is ultimately, it is claimed, to experience literature.

### The heroic philosopher as a non-reader

When, in 1887, Friedrich Nietzsche (1968: 47) identified “the reader” alongside “the historian,” “the critic” and “the collector” as a “*reactive* talent,” he was articulating a long-held suspicion among philosophers about the figure of the reader—as too passive, too dependent. Hence, in a parallel fashion, two centuries earlier René Descartes inaugurated the image of the modern philosopher by *throwing out books*: according to Descartes (1988: 26), the philosopher achieves maturity by substituting her dependence on “the sciences contained in books” with “the simple reasonings [of] a man of good sense, using his natural powers.” As such, in Descartes’ *Meditations on First Philosophy*, proper thinking (i.e., meditation) only occurs once the words themselves have fallen silent, once the writer “pauses” at the end of an argument and asks the reader to “contemplate” free from the text (Descartes, 1988: 98).

While these are extreme examples,^9^ variations on this principle of the non-simultaneity of reading and thinking (i.e., the principle that thinking properly occurs *after* or *away from* reading) is common in modern Western philosophy. Consequently, it has become relatively rare for reading to become an object of philosophical attention at all.^10^ Outside of small pockets of the history of philosophy, philosophers like to think of themselves as active thinkers rather than passive readers, and so those who *merely* read are taken as an abject foil for the figure of the philosopher: as grammarians, philologues, scholars or historians. This aversion to the seeming heteronomous position of the reader (who is hospitable to the words of others) is, in part, an expression of an aversion to dependence on material objects, particularly the contingencies of a physical book.^11^ When Kant refuses to “make my head into a parchment and scribble old, half-effaced information from archives on it” (Kant, quoted in White Beck, 1963: vii), he expresses a widespread fear of the archive that seems to be but one more manifestation of a fundamental axiom: the philosopher should not read too much in case she thereby stops thinking.

### Reading as interpretation

In those relatively rare instances when philosophical reading does become an object of philosophical attention, it is understood as a purely interpretative act, a series of hermeneutic operations that produce meaning. Philosophers, just like many literary critics “share an implicit assumption that reading is a synonym for interpretation” (Auyoung, 2020: 93), that it is nothing but sense making. Hence, there might be periodic controversies over whether philosophical reading should be practiced as deconstruction, rational reconstruction, hermeneutics, symptomatic reading, and so on; but all of the theories in dispute are ultimately variations on interpretative method. As Nussbaum (1992: 62–63) points out in a slightly different context, this view has “powerful roots in an entire intellectual tradition” according to which “passions and our feelings are unnecessary to the search for truth” and so according to which “a discourse that claims to search for truth and impart knowledge must speak in the language of the intellect.” The reduction of reading to interpretative operations rehearses, indeed, a version of Cartesian dualism, in which the psychological and physiological sensations experienced in reading are suppressed in the name of the textual object’s status “as a transparent vehicle for the meaning and interpretative acts [that exist] *for* consciousness” (Littau, 2006: 24). Successful reading “lifts the reader from sensation to intellect” (More, quoted in Littau, 2006: 5), such that the text becomes purely “an occasion for interpretation” (Tompkins, 1980: 206). In Wimsatt and Beardsley’s terms, the reader is little more than an “explicator of meanings” (Wimsatt and Beardsley, 1949: 48). As with the previous two “pathologies,” there is (exempting some specialist practices in the history of philosophy) something of an anxiety toward *textual material* behind this treatment of the act of reading, a loss of “the physicality of reading” (Littau, 2006: 2).^12^

Wimsatt and Beardsley’s (1946) rubric of “the affective fallacy” is precisely intended to justify the above reduction of reading to interpretation (in parallel to their rubric of “the intentional fallacy”). Whereas the intentional fallacy stands as a criticism of any reading determined by authorial origins, the affective fallacy is intended to criticize any conclusions drawn from the text’s readerly effects. They write, “The Affective Fallacy is a confusion between the poem and its *results* (what it *is* and what it *does*)… It begins by trying to derive the standard of criticism from the psychological effects of the poem and ends in impressionism and relativism” (1949: 31). As this suggests, there is more at stake here than just the validity of emotional responses to texts; for one, it is also a question of the dominance of interpretation in twentieth- and twenty-first-century accounts of reading. As Sontag (1966: 97–99) argued, when rational reconstruction becomes so hegemonic as to turn into pure translation, this is tantamount to “an open aggressiveness” toward—and “overt contempt” for—the text itself. Taken to extremes, “to interpret is to impoverish.”

Even more fundamentally, what is at stake in any interrogation of the cogency of “the affective fallacy” for determining how philosophy is read is some sense of the text *doing something*. The reduction of reading to interpretation omits the effects of the philosophical text. As Tompkins (1980: 222, 225) writes, “Once the… work has been defined as an object of knowledge, as meaning not doing, interpretation becomes the supreme critical act… The text remains an object rather than an instrument, an occasion for the elaboration of meaning rather than a force exerted upon the world.” For those who insist on the affective fallacy, reading philosophy is solely a task of extracting meaning from an artifact. Nevertheless, this is, Tompkins argues, a contingent, limited perspective: in other traditions and other epochs, texts were treated “as a force acting on the world, rather than a series of signs to be deciphered.” (1980: 203).

Some of what I am trying to get at here can be briefly developed by means of Claudine Tiercelin’s comments on the development of “the idea” in the pragmatist tradition. For someone like Arthur Lovejoy, according to Tiercelin (2020: 2–3), an idea is less determined by its truth, its consistency or even the subtleties of its meaning than by what an encounter with such an idea *does*—its effects on the reader, on the philosophical community and on the philosophical tradition. What matters is the “practical force of realization” of ideas, “their efficacity and their force, rather than their coherence or internal logic.” The perspective that emerges is one in which works of philosophy are not only static artifacts to be deciphered, but also historical processes formed by collisions with readers (2020: 6).

## Lay readers and professional readers

This is not to say that professional philosophers never speak of works of philosophy being fun or boring. They do, but only within the strictly confined role of *stimulus*, i.e., as an initial provocation to philosophical reflection that will ultimately be superseded by the demands of rational reconstruction. That is, the philosopher might use an experience of reading as a propaedeutic to doing “proper” philosophy—much like Kant (2011: 86) who needed to keep reading Rousseau so that “the beauty of his expressions no longer disturbed me, and only then could I finally examine him with reason.” Such a strategy is most visible in the classroom: that students found a piece of philosophy annoying, fatiguing or (very occasionally) fun serves as an icebreaker that opens up a dialectic intended to propel the participants into more rarefied philosophical regions, i.e., those regions academic philosophers value more highly. At its most extreme, this can become a presumption that the role of a philosophy-education is to train students out of these “improper” experiences of reading through a discipline that aims at forming impassive, “cerebral” philosopher-readers.

Implicit here is a distinction between “professional” and “lay” philosopher-readers, and, consequently, a distinction between “high” and “low” reading practices. Professionals in philosophy departments are counted on to read disinterestedly, because they have, it is assumed, internalized some disciplined habit of reading that abstracts them from the claims of the immediate and the passionate—that is, through the cultivation of a readerly *ataraxia*. Two conclusions follow. First, this professional image of the philosopher-reader reflects the experience of a very specific subject with very specific gender, ethnicity and class commitments. At the very least the values coded into this kind of reading practice constitute a good example of what Genevieve Lloyd (1993) identified as “the historical maleness of reason.” Secondly, this professional image of the philosopher-reader is not the only one possible; there are plenty of other types of philosopher-reader out there. When David Conceptión (2019) admits that, as a student, “I did not know how to read philosophy… I did not know how to read as philosophers read,” what seems significant is less the implication that his student self was somehow ignorant or wrong than the implication that his younger self read according to other goals, conventions and forms of enjoyment than the professional ones. As Auyoung (2020: 93) puts it in the context of literary criticism, “We [as professionals] have sought to establish a radical yet largely tacit discontinuity between our reading practices and those of non-specialists, including members of other academic disciplines, who may think they are reading but cannot really read at all.” The professional philosopher has, in other words, happily taken on the role of policing what “counts as real reading” (2020: 94)—and this is fundamentally what is at issue in the first half of the present essay: we, as professional philosophers, tend to take for granted what reading philosophy looks like and make use of this tacit definition in a fairly exclusionary manner.

Nevertheless, perhaps a more constructive way of articulating the disconnection between professional and lay philosopher-readers is not so much in terms of a break between different types of reading practice than in terms of how professional philosophers perform and present their reading practices to others (whether in a classroom or a journal). That is, professional philosophers might encounter texts in all sorts of ways, but the way in which they formally relate these encounters is strictly regulated and so homogenized. Auyoung (2020: 95) again provides a useful gloss on “how critical reading is represented”: “Far from capturing the messiness and multiplicity of their actual experiences of reading, [in publications] critics construct coherent arguments by presenting an extremely limited selection of the inferences they have made during the reading process.” These constraints on the professional representation of reading thus leave open the possibility of silent, but shared experiences of reading philosophy that have not been deemed worthy of representation in professional philosophy. In other words, all philosophers might get excited, bored or seduced by philosophical writing, but we just do not hear about it in academic journals. And yet, Davis (1991: xv) reminds us, “It is not good for us to feel ashamed of what we naturally do when we read.”

This is no place to set out a wholesale sociology of academic philosophy. Nevertheless, it is worth conjecturing that, just as cultural historians (e.g., Radway, 1991) have long been describing literary reading-communities within and outside the academy and making visible the very different reading habits on display there, further research on the structures and practices of philosophy reading-groups, philosophy cafés and “philosophy in pubs” meetings that occur outside the academy would provide a fuller picture of what the experience of reading philosophy looks like.^13^ Further pedagogical research might also shed light on the role of affect in the learning of philosophy-students, especially outside of the classroom, in study groups, etc. There is more than one community of philosopher-readers, and, indeed, one of the more obvious features of the above examples is their communal character, as opposed to professional reading practices often oriented toward individual research projects. The self-denying community of the modern research university^14^ stands in contrast to concrete communities of shared reading. In fact, it seems clear that any full account of experiences reading philosophy would need to describe the panoply of “goals,” “institutions,” “communities,” “initiations,” “disciplines,” and “ideals” that determine them.^15^

## Epistemic breakthroughs

In addition to the above high-level description of the structures of reading philosophy, there is space for a personal approach to the singular moments that constitute these experiences. And this is what I want to begin to explore in the final pages of this essay.

`There is a mystery in reading,’ writes Simone Weil in opening her `Essay on the Concept of Reading’ (Weil, 2015: 21). Such a the `mystery’ concerns the discrepancy (or `contradiction’, according to Weil) between what reading seems to involve–`some black marks on a sheet of white paper’–and what it can bring about–something comparable to `a punch in the stomach’. She writes, `Sometimes a combination of novel signs that I have never seen seizes my soul right where the wounding meaning penetrates, along with the black and the white, and just as irresistibly.’ That is, in the event of reading, a meaning can strike us as viscerally as a violent sensation, along with all its physiological and psychological effects: sensation and meaning are indistinguishable; both directly “jump out” from the text. (2015: 21-2, 25) A useful example of such a sensation experienced in an act of philosophical understanding is furnished by A. S. Byatt in *Still Life* (Byatt, 2003). The protagonist Stephanie returns to an academic library after a hiatus in order to get some reading done; she attempts to get back in the flow of thinking among books, and, amidst everything else going on, she successfully experiences a surge of insight once more:

[Stephanie] remembered the sensation of *knowledge*, of grasping an argument, seizing an illustration, seeing a link… Knowledge had its own sensuous pleasure, its own fierce well-being, like good sex, like a day in bright sun on a hot empty beach. (2003: 185; discussed in Davis 1991: 40–42)

Byatt’s Stephanie is caught up in the attempt to give shape to (and recall) what it feels like to gain knowledge—a sensation that encompasses far more than the sum of its interpretative acts. There is pleasure, physical contact, warmth and even peace involved in Stephanie’s feeling for getting at a truth while reading. And this, I want to claim, holds true of any successful attempt at reading philosophy as well: it comprises a “sensation,” a bundle of thoughts and feelings, interpretative operations, personal transformations, discoveries and passionate encounters, all of which pertain to some primitive surge of insight. Such a bundle is not composed of distinct, successive or independent elements, but rather everything is fused in an “aha-moment,” an event of thinking erupting with in the act of reading.^16^

Sophie Grace Chappell’s recent study of the structure of epiphany provides a helpful framework for the sensations involved in this kind of readerly epistemic breakthrough. Chappell (2019: 102) is quick to note that the concept of epiphany need not be limited to epistemic breakthroughs alone, it can be more or less cognitively loaded—in her terms, more “wow-moment” than “aha-moment” (see further Chappell, 2022). However, for the purposes of my interest in *philosophical* epiphany, “aha-moments” provide the paradigm for what it feels like to experience some kind of cognitively-loaded breakthrough while reading philosophy, i.e., what it feels like to experience a truth when reading and to stay faithful to that truth. Chappell (2019: 97) goes on to list some of the features that might be attributed to this type of readerly insight: “overwhelming,” “existentially significant,” “often sudden and surprising,” the bringer of “something new,” “something given, relative to which I am a passive perceiver.” These epiphanies can take the form of “a peak of delight; or of vividness; or of forcefulness or intensity; or of lucidity; or of horror, or of terror, or of anger” (2019: 98). Generally, they constitute “sharp,” peak encounters with meaning and so contrast with more everyday meaningfulness (2019: 98–9). As Chappell (2019: 104) also notes, epiphanies are not necessarily private, but can be public and communal, as well as (initially, at least) subconscious, such as when a reader is taken unawares by a new way of seeing. In general, she emphasizes the extent to which any account of these epiphanic moments must be attentive to their “broad and open-edged, and even messy” nature (2019: 104).

Amidst this panoply of characteristics that philosophical epiphanies might display, there are two particular kinds of epistemic breakthrough I want to focus on and which, in many ways, recapitulate some of my earlier discussion in this essay—that is, on the one hand, those breakthroughs that take the reader away from the text and, on the other, those that keep the reader immersed within it, i.e., thinking-in-reading.

### Breakthroughs away from the text

The first kind of readerly epiphany has already been described in a number of forms in the foregoing: it constitutes the moment at which something catalytic in the philosophical text propels the philosopher into a thinking beyond reading. The Cartesian principle of the non-simultaneity of reading and thinking forms the basis for such a model: reading serves as a propaedeutic to thinking, a ladder that falls away once the philosopher finds herself initiated into a problem, an argument or a debate. Philosophers read in order to leave reading behind, i.e., attain the point at which the text—with all its contingencies and idiosyncrasies—stops getting in the way.

A pertinent example of this structure is provided by the “philosophy as a way of life” movement. This is because philosophy as a way of life shares many of the concerns I have been discussing above: a distrust of professional philosophers and their monopoly on representing how philosophy is done [what Hadot (1995: 270) disparagingly calls the perennial structure of “professionals training professionals”]; an interest in the contribution lay-philosophizing might make to the discipline (see Sellars, 2017: 48); and a corresponding emphasis on the transformative effects of the pursuit of wisdom when undertaken in all sincerity. And yet, endorsements of philosophy as a way of life are typically accompanied by a denigration of the practices of writing and reading philosophy—based on the principle that “actions are ultimately more philosophically significant than words” (Sellars, 2017: 41). As Shusterman (1995: 40–41) notes, philosophy as a way of life has “asserted itself as something other and more than textual exercises,” as a set of transformative practices that occurs “beyond mere utterances of textual inscriptions.” The figure of Socrates looms large here as a philosopher who neither read nor wrote, but acted out philosophy.^17^ In other words, reading is not enough; its purpose is solely to provoke the philosopher to become more than a reader. On the one hand, this means that the philosophy as a way of life movement is also interested in dismantling the myth of the affective fallacy, so as to show how philosophical texts “actually *do* something to the reader,” i.e., “immediately affect, touch, concern, disturb, intrigue, provoke, and make one angry, or else attract, seduce, entice, stimulate, inspire, and obsess the reader” (Faustino, 2020: 368). Nevertheless, on the other hand, according to this model, philosophical writing *acts on* the reader, so as to put an end to mere reading; texts function as instruments for disclosing the more-than-textual. The principle of the non-simultaneity of reading and thinking remains operative.

### Thinking-in-reading

In the midst of my “off-duty” reading of the *Critique of Pure Reason* described in §1, I imagine stumbling across a footnote in the chapter on the antinomies which I barely remember having read before. It begins, “To the question, “What kind of constitution does a transcendental object have?” one cannot indeed give an answer saying what it is, but one can answer that the question itself is nothing” (1998: A478/B506). This sentence gives me a jolt: there is something about Kant’s attack on the very asking of the question, “what is a transcendental object?,” that makes me think. The emergent spark is not particularly well-formed as yet, but it broadly concerns how many Kant scholars still seem to ask the kinds of questions Kant himself seems to want to disqualify with this comment. However, rather than sending me off into the scholarship or even into a reverie about the subtleties of transcendental idealism, this is a surge of inchoate insight that makes me impatient to turn back to earlier sections of the first *Critique*, to reread parts of the Transcendental Aesthetic and Transcendental Logic from this new standpoint (according to which Kant’s use of the language of questioning is in some way significant). I am excited to get to grips with my surprise realization, to test it and to develop it by reading more, by getting further into the first *Critique*, i.e., by doing philosophy within the words of the book in front of me. In short, as a result of this event in reading, *the Critique of Pure Reason* now looks different to me: it strikes my attention in a different way, with a new texture, a new topography.^18^

This is another model for an epistemic breakthrough in philosophy, one in which reading more and reading better serves as thinking, in which, that is, thinking takes on the form of passionate reading. It is here we find some preliminary clues as to the “sensation of knowledge” involved in the experience of reading philosophy—and, as might be expected, this sensation splinters into a number of variations:

#### Thinking-in-reading as force

My encounter with Kant’s remark on the question of the transcendental object late in the first *Critique* propels me, as reader, backwards through the text, as well as deeper into it. My personal discovery in an out-of-the-way footnote acts on me, reshapes my understanding and redetermines my route through the text.

#### Thinking-in-reading as investment

Kant’s emphasis on the “nothingness” of the question of the transcendental object demands a new kind of reading; it gives rise to reading habits that are more active, more invested: I’m now intent on making my new idea work when it comes to the first *Critique* as a whole. In other words, much more is now at stake for me in my reading.

#### Thinking-in-reading as becoming an insider

An illumination that comes from out of nowhere instantly transports me, as reader, into a new logic of the text, into a new appreciation of its patterns and structures, whereas before I had felt like an outsider. This is an unexpected instant of “getting it” (even if it ultimately gets me nowhere) provoked by a previously invisible configuration of terms.

#### Thinking-in-reading as a minimal disturbance

For many other readers, this footnote reveals nothing: it tells them little that is new and certainly nothing that takes on disproportionate significance for them. But, for me, Kant’s (1998: A478/B506) claim that the question of the constitution of the transcendental object is “empty and nugatory” makes me think differently. It acts as some small bump at an unassuming moment within the text with far-reaching consequences for how I in future will encounter the first *Critique*.

#### Thinking-in-reading as tact

The different reading practice that emerges out of this realization is non-linear: I move through the first *Critique* according to different concerns and, as a result, become a more mobile reader. I glide (insofar as I’m able) against the flow of arguments, between otherwise disconnected concepts, to discover new resonances.

These are but five ways in which one imagined experience of doing philosophy can be rendered, five ways of describing a surge of insight experienced while reading, however small and ultimately fruitless such an insight might turn out to be.^19^ Nevertheless, these descriptions help tell us something about what we do when we do philosophy, and so contribute to an increasingly complete description of what it feels like to be a philosopher.

### The enjoyment of frustration

The *Critique of Pure Reason*’s Transcendental Deduction is, according to Kant (2004: 4:260) himself, “the most difficult thing that could ever be undertaken on behalf of metaphysics.” And most readers agree. The difficult reputation of the B-version of the Deduction, in particular, strikes fear into most who venture into it: Shaddock (2013: 155) is surely right to gloss Kant’s comment, “Readers have found it not just difficult but downright impossible.” More readings of the Transcendental Deduction end in failure than success; that is, if Kant constructs a language that is “painful to understand” for his readers (as Joubert put it), then the Deduction is where that pain is felt most acutely. And yet, readers keep coming back to this section of the first *Critique*; we are gluttons for punishment. The pain of reading the Deduction is something philosophers seem to want to experience repeatedly, like some cerebral extreme sport. Kant (1989: 5.334) will elsewhere speak of books “that break your head” and books “that break your neck,” as well as the books “that break your heart” by sentimental novelists. One lesson to learn from the above is surely that some philosophical explanation is required for the fact that philosopher-readers enjoy failing, despite it all, that they have a passion for headaches.

Philosophers are distinctive insofar as they enjoy failure. The philosopher-reader is someone who spends a lot of time being frustrated at *not “*getting-it,” at *not* experiencing some epistemic breakthrough. In other words, while readers often turn to philosophical texts to make themselves feel better, they also turn to philosophical texts *to enjoy making themselves feel worse*. This “feeling worse” comes in two forms: first, readers might turn to philosophy to exacerbate or even generate anxieties about meaning, knowledge, or particular human values; secondly, readers might turn to philosophy *in order to be puzzled*, to fail to understand the text being read. On this second kind of reading, the reader encounters something in the text that acts as a barrier to epistemic or existential breakthrough, a blockage that lends itself to frustration and alienation, instead of epiphany and insight. This is, in many ways, a constitutive characteristic of the philosopher-reader: situating oneself as an outsider trying and failing “to get in,” positioning oneself as alienated from the order of philosophical reasons contained in the text and, therefore, engaging in an antagonistic and doomed battle with the author’s words to ultimately gain access to these reasons. It is something of what Conceptión (2019) intends when he speaks of “the strangeness and disquiet that so often comes with reading philosophy.” This is, in short, a sort of philosophical reader’s block that leaves its mark on a lot of philosophical practice.

Any account of the experience of reading philosophy should not just involve, therefore, descriptions of what it is like to encounter a truth; it should also contain descriptions of what it is like to enjoy the messy combination of positive and negative passions felt whenever ideas refuse to come, whenever a breakthrough does not occur and whenever, nevertheless, one keeps coming back for more.

## Conclusion: Reading and the philosophical life

A phenomenology of the philosophical life (whether professional or otherwise) must include a description of what happens when we read philosophy. Reading is not a peripheral philosophical pastime (i.e., what we do when we tire of thinking for ourselves); it constitutes much of what we do when we do philosophy. To ignore the experience of reading is both to ignore what most philosophers are up to for the majority of their time and to ignore one of the most significant forms that philosophical thinking can take—*thinking-in-reading*. Moreover, once one does start paying attention to this experience of reading philosophy, it soon becomes clear how much more it involves than just interpretative acts: the philosopher-reader is subject to, among other things, sensations, passions, emendations and transformations. A fuller account of philosophical reading should therefore outline some of the sociological structures that determine how different communities of philosophers (within and outside the academy) construct such experiences, as well as describe in detail ways in which philosophers encounter (or fail to encounter) truths while reading. More generally, any fuller account should describe ways in which philosophy acts upon readers and the various effects that result.

## Data availability statement

The original contributions presented in the study are included in the article/supplementary material, further inquiries can be directed to the corresponding author.

## Author contributions

The author confirms being the sole contributor of this work and has approved it for publication.

## Conflict of interest

The author declares that the research was conducted in the absence of any commercial or financial relationships that could be construed as a potential conflict of interest.

## Publisher’s note

All claims expressed in this article are solely those of the authors and do not necessarily represent those of their affiliated organizations, or those of the publisher, the editors and the reviewers. Any product that may be evaluated in this article, or claim that may be made by its manufacturer, is not guaranteed or endorsed by the publisher.

## Footnotes

^1^In what follows, I make considerable use of (sometimes relatively old) sources from literary theory and criticism, because, I argue, philosophy’s relation to reading constitutes a *missed opportunity* (in a way that of the study of literature does not). Philosophers too should pay attention, in Roland Barthes’ (1977: 148) words, to "the birth of the reader." See §2 below on the risk of this (apparent) making-literary of philosophy.

^2^I return to this passage in §2 below.

^3^There are of course significant differences between the experience of reading a philosophy text for the first time and the experience of repeated readings that I leave aside for a future occasion.

^4^I take these ideas of competing readerly selves and "off-duty" readings from Pearce (1997: 3).

^5^I could equally, of course, have chosen some contemporary work of philosophy with similar results. It is also worth emphasizing from the beginning that I have chosen Kant’s *Critique of Pure Reason*, rather than any text more closely situated to the borderline between philosophy and literature (e.g., Nietzsche, Camus), precisely because of Kant’s attempts to avoid both popularity and conscious writerly effects. Even a book as seemingly abstract and scholastic as the *Critique of Pure Reason* can provoke howls of passion. More than many other philosophers, there is a visible disjunct between Kant’s (apparent) cultivation of passionless neutrality and the affect-laden responses of his readers. For one thing, this hopefully suggests from the beginning that to talk about various passionate experiences of reading philosophy is *not* to necessarily talk about the *writing of philosophy*. The philosopher-reader is a figure of interest independently of the philosopher-writer.

^6^This language is taken from Sophie Grace Chappell—see §4 below.

^7^I speak of "modern" philosophy in particular, since the pre-modern relationship between philosophy and eloquence seems to give rise to a closer connection between doing philosophy and "moving" an audience. There are obviously exceptions to these generalisations, such as, in the twentieth century, Ferdinand Alquié (1979, 2005), for whom the encounter with past philosophical texts requires an engagement from "affective consciousness".

^8^Generally, within the essays collected in *Love’s Knowledge* Nussbaum establishes an opposition between, on the one hand, the philosophical *reading of literature*, which can be passionate, open, "finely aware and richly responsible" (quoting Henry James; see Nussbaum, 1992: 140), and, on the other hand, the philosophical *reading of philosophy* which remains "retentive and unloving" (1992: 282). The philosopher-reader learns from literature how to be "keenly alive in thought and feeling to every nuance of the situation, actively seeing and caring" (1992: 143), to be "humble, open, active, yet porous" (1992: 282). But this learning remains constrained to literary material. Whenever we encounter a straightforwardly philosophical text, "we aren’t very loving creatures" (1992: 282). That is, Nussbaum to some extent denies philosopher-readers an enriched experience of *philosophical* texts.

^9^Other extreme examples are not hard to dig up—for example, anglophone common sense philosophy. As Hare put it in, "We do not think it a duty to write books; still less do we think it a duty to read more than a few of the books which others write." (Hare, 1960: 113–14) See the discussion in Rée (1993: 11–12).

^10^This stands in contrast to the repeated attention paid to philosophical *writing* and the figure of the philosopher-writer. The various ‘philosophy as literature’ movements (whether post-Danto or post-Derrida) typically consider philosophy in terms of authorial choices concerning the most appropriate textual form for disclosing a thought. What is at stake are the origins of a philosophical text, rather than its effects.

^11^Smith (2016: 12) puts this nicely: contemporary philosophers "are not interested in thinking about the way in which we deploy standards of evidence when considering textual sources, or secondary testimony, or other such philological matters. To take an interest in these questions would be to acknowledge that philosophy has a philological component, and therefore cannot be, simply, an unmediated, eternal conversation. [It forms part of] the general refusal to consider the discipline as in part a philological endeavor." Moreover, perhaps the most relevant conclusion to be drawn from Smith’s history of the figure of the philosopher in general (Smith, 2016) is that, of the six different images of being a philosopher he describes, none of them spend much time reading.

^12^Nevertheless, the readerly materiality that makes philosophy possible does sometimes manifest itself. In the final section of his *Conflict of the Faculties*, Kant (1979: 189) considers the barriers that the physiological constitution of the human might impose on doing philosophy. He is not unduly worried by most of them, since, in general, "mental work can set another kind of heightened vital feeling against the limitations that affect the body alone," even if the fact that philosophers have a body still ultimately requires "a diet with regard to thinking" (1979: 199). However, of those afflictions that cannot be regulated by mental powers but instead "impede thinking," the one with which Kant (1979: 207) concludes the work concerns the manner in which "our eyes are harassed from all sides by the wretched affectations of book printers." That is, philosophy cannot take place without ‘the protection of the reader’s eyes’ from the material appearance of books. Kant (1979: 208) himself describes suffering attacks in which, "when I am reading, a certain brightness suddenly spreads over the page, confusing and mixing up all the letters until they are completely illegible." It is for this reason he sets guidelines for typesetters (1979: 208) to improve the physical condition of books on which his work as a philosopher depends.

^13^For some comments around this issue, see Stern (2015).

^14^See Whistler (2016), which in turn builds on Howie (2002) and Howie and Tauchert (2005).

^15^In other words, to provide such an account the philosopher would need to start describing doing philosophy from a perspective "thickened" by the methods and conclusions of (for example) anthropology, sociology, autoethnography and literary studies. That is, philosophy as a discipline tends to be blind to the social and cultural formation of its own practices and a more convivial, multidisciplinary approach would, in part, begin to remedy this.

^16^Even the most apathetic of professional philosopher-readers still reads intent on experiencing some version of this bundle of cognitive, affective and emendative effects, for, to return to Kant (1989: 5.272), "*Even being without affects* (*apatheia*, *phlegma in significatu bono*)… is sublime" and is caught up in "pure reason’s liking."

^17^ Nietzsche’s *Schopenhauer as Educator* has also proven a fortuitous source-text in this regard. Nietzsche is clear, "The philosopher must supply [an] example in his visible life, and not merely in his books; that is, it must be presented in… facial expressions, demeanor, clothing, food, and custom more than through what they said, let alone what they wrote." He continues, "Critique… has never been taught at universities: all that has ever been taught is a critique of words by means of other words." (Nietzsche, 1995: 183-4)

^18^What is not discussed here—and exceeds the scope of the present essay—is any reference to the sharing of such experiences, in terms of both strategies of communicating them to others and the ways in which such dissemination influences and determines further reading experiences. To put it another way: the ‘I’ of this fiction is certainly not the first person to have the above insight when reading the first *Critique* and knowledge of this must shape their reading experience.

^19^For further discussions of these kinds of descriptions, see Davis (2020: 5).
